# Magnetophotoluminescence of Modulation-Doped CdTe Multiple Quantum Wells

**DOI:** 10.1021/acsomega.3c05546

**Published:** 2023-10-16

**Authors:** Wiktoria Solarska, Krzysztof Karpierz, Maciej Zaremba, Florian Le Mardelé, Ivan Mohelsky, Adam Siemaszko, Mikołaj Grymuza, Łucja Kipczak, Natalia Zawadzka, Maciej R. Molas, Eryk Imos, Zbigniew Adamus, Tomasz Słupiński, Tomasz Wojtowicz, Milan Orlita, Adam Babiński, Jerzy Łusakowski

**Affiliations:** † Faculty of Physics, University of Warsaw, L. Pasteura 5, 02-093 Warsaw, Poland; ‡ Laboratoire National des Champs Magnétiques Intenses, CNRS UPR3228, EMFL, Université Grenoble Alpes, Université Toulouse, Université Toulouse 3, INSA-T, F-38042 Grenoble and Toulouse, France; § International Research Centre Mag Top, Institute of Physics, Polish Academy of Sciences, Al. Lotników 32/46, 02-668 Warsaw, Poland; ∥ Institute of Physics, Polish Academy of Sciences, Al. Lotników 32/46, 02-668 Warsaw, Poland; ⊥ Centera Laboratories, Institute of High Pressure Physics, Polish Academy of Sciences, Ul. Sokołowska 29, 01-142 Warsaw, Poland

## Abstract

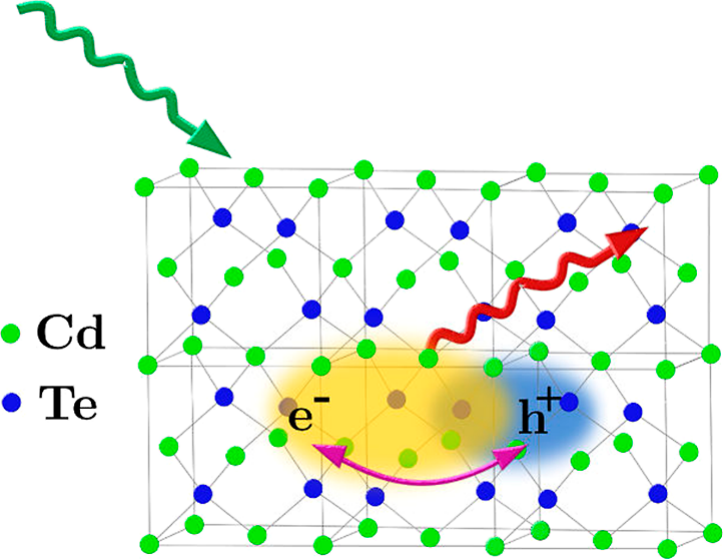

Modulation-doped CdTe quantum wells (QWs) with Cd_0.7_Mg_0.3_Te barriers were studied by photoluminescence (PL) and far-infrared Fourier spectroscopy under a magnetic field at 4.2 K and by Raman spectroscopy at room temperature. Two samples were tested: a sample which contained ten QWs (MQW) and a sample with one QW (SQW). The width of each QW was equal to 20 nm, and each of them was modulation-doped with iodine donors introduced in a 4 nm thick layer. The concentration of donors in each doped layer was nominally identical, but the thickness of the spacer in SQW and MQW samples was 20 and 10 nm, respectively. This resulted in a two times higher electron concentration per well in the MQW sample than in the SQW sample. We observed differences in PL from the two samples: the energy range of PL was different, and one observed phonon replicas in MQW which were absent in the SQW sample. An analysis of oscillations of the PL intensity as a function of magnetic field indicated that PL resulted from the recombination of free electrons in the conduction band with free or localized holes in the case of SQW and MQW samples, respectively.

## 1 Introduction

Optical properties of CdTe quantum wells (QWs) with (Cd, Mg)Te barriers have been studied since their fabrication in 1990s.^1−4^ The advantage of this family of II–VI quantum structures is related to relatively large band offsets and a small lattice mismatch between CdTe and (Cd, Mg)Te which leads to structures of high crystallographic quality. Photoluminescence (PL), mainly in magnetic fields, Raman scattering, and time-resolved or optically detected resonance measurements were carried out on structures with different sequence of layers, doping, or grown on different substrates to characterize properties of quasi-particles (electrons, holes, excitons, etc.) in these materials.

An important part of this research concentrated on the influence of a two-dimensional electron gas (2DEG) on PL. Introduction of free electrons into QWs was achieved either by doping of the wells or by a modulation doping when the doped region of the barrier is separated from the well by an undoped spacer. Modulation doping allows one to control the concentration of free electrons in the well *n*_c_ in a broad range (typically between 10^9^ and 10^12^ cm^–2^) and allows the separation of free electrons in the well from their host donors which leads to a higher quality of a 2DEG resulting from a lower rate of scattering by impurities.

It is interesting to note that the 2DEG concentration can be determined not only by transport measurements but also by optical methods. For example, to this purpose, an analysis of polarization-resolved reflectivity under a magnetic field from a CdTe/(Cd, Mg)Te sample in the energy range of trions’ excitation was applied in ref (5), while a nonresonant THz photoresistance was used in ref (6). On the other hand, another basic property of a 2DEG, namely the electron *g*-factor, can be determined by means of a spin-flip Raman scattering.^7^

In nominally undoped QWs, excitons are observed, while trion-related lines^8^ appear to be the strongest feature at moderately doped samples (*n*_c_ ∼ 10^10^ cm^–2^). At still higher *n*_c_, above 10^11^ cm^–2^, the PL spectrum evolves toward a broad maximum dominated by a Fermi edge singularity (FES). An experimental evidence of such an evolution of the PL spectrum was presented in ref (9), and a theoretical analysis was given in a few papers.^10,11^ However, under a strong magnetic field, FES evolves into trions,^12^ and a recombination of dark triplet trions becomes the most intense line in the PL spectrum.^13^ Another type of excitation related to the presence of a 2DEG in QWs is a combined exciton–cyclotron resonance^14^ in which an incident photon creates an exciton and simultaneously transfers an electron between neighboring Landau levels.

Advanced experimental methods, including pulsed techniques, have also been applied to describe spin-dependent phenomena in CdTe/(Cd, Mg)Te structures.^15−17^

In the present paper, we compare the low-temperature PL from two samples with CdTe QWs and Cd_0.7_Mg_0.3_Te barriers. There is one QW in the single QW sample (SQW) and ten QWs in the multiple QW sample (MQW). In both samples, all wells are 20 nm wide, and each of them is modulation-doped with iodine donors. To analyze the spectra, we applied a method proposed by Babiński et al.^18,19^ which consists of an analysis of oscillations (as a function of magnetic field) of PL intensity in narrow-energy ranges covering the PL spectrum. The analysis allowed us to show that in the case of the SQW sample, PL can be interpreted as resulting from a recombination of free electrons and free holes with a wave function which is a mixture of light and heavy hole states. On the contrary, in the case of the MQW sample, holes are localized. Also, the spectral range of PL in the case of the two samples is different, and phonon replicas of PL are observed in the case of MQW sample only. We discuss possible reasons of these differences pointing at the role of the electric field, a nonzero wave vector of recombining particles, and the influence of localization potentials resulting from the fluctuation of the electrostatic potential.

## 2 Samples and the Experimental Setup

**Figure 1:**
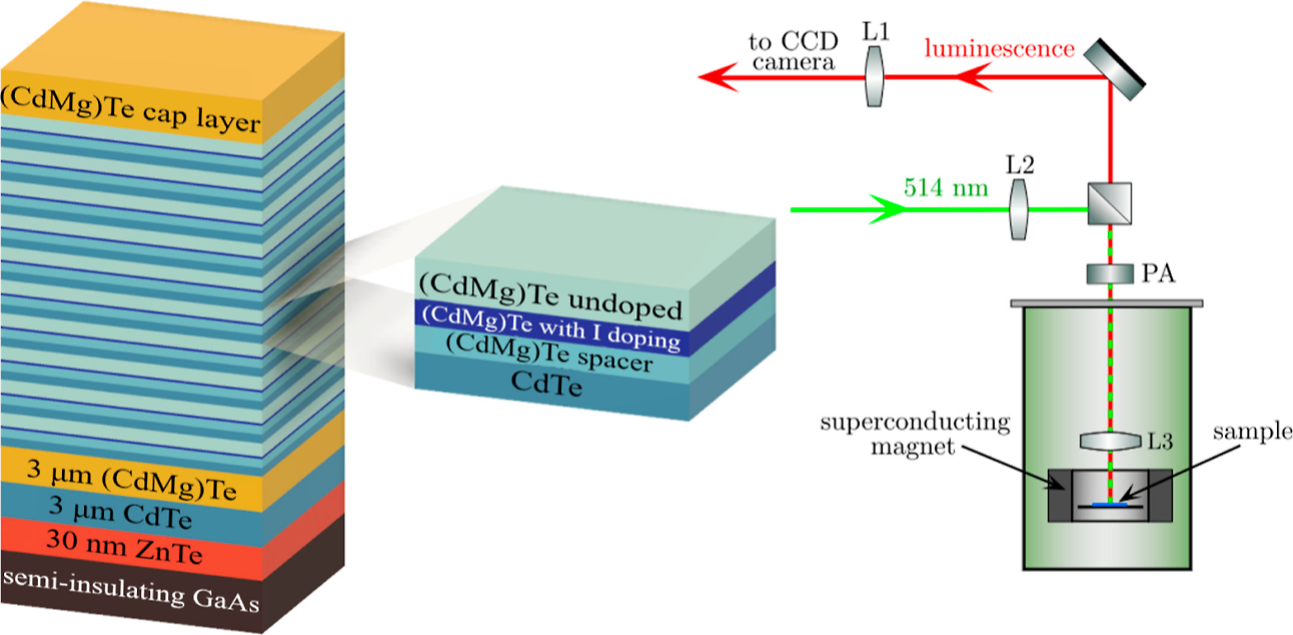
Scheme of the MQW sample’s structure (left) and a scheme of the experimental setup (right). The focal length of the lens L1 is equal to 5 cm, while those of L2 and L3 are 3 cm. In reality, the lens L3 is inside the magnet. PA—the polarization analyzer.

The samples used in the present experiment were modulation-doped CdTe QWs with Cd_1–*x*_Mg_*x*_Te barriers grown by molecular beam epitaxy. A semi-insulating GaAs wafer served as a substrate; the magnesium content *x* in all (Cd, Mg)Te layers in the structures was kept constant and equal to *x* = 0.3. Buffer layers [ZnTe, CdTe, and (Cd, Mg)Te] were grown to decrease the lattice mismatch between the substrate and QWs. In each case, the width of a QW was equal to 20 nm, and the thickness of a modulation-doped layer (iodine donors) was 12 monolayers (i.e., about 4 nm). The temperature of the iodine source was the same during the growth of both samples, which resulted in the same concentration of iodine donors in the doped layer. However, the spacer in SQW and MQW samples was 20 nm and 10 nm thick, respectively, which resulted in the electron concentration equal to 4.8 × 10^11^ in SQW and 1.0 × 10^12^ cm^–2^ in each MQW. In the MQW sample, the distance between neighboring wells was equal to 44 nm (10 nm of spacer + 4 nm of doping layer + 30 nm of undoped barrier). A scheme of the MQW sample and the experimental system is shown in Figure 1.

The samples were mounted in an insert (a stainless-steel tube of length of about 1.3 m) and positioned at the center of a superconducting magnet, generating a magnetic field of up to 9 T. They were cooled with an exchange gas at a pressure of a few mbar to the temperature of 4.2 K. The luminescence was excited with an Ar^+^ multiline laser light (two main components were 488 and 514 nm), spectrally dispersed by a spectrometer and detected with a CCD camera. An analyzer of the circular σ^+^ and σ^–^ polarizations was placed in the optical path between the beamsplitter and the inset to allow registering polarization-resolved spectra. The circular polarizer used was composed of an achromatic λ/4 waveplate and a linear polarizer that polarized only the luminescence (around 770 nm) and not the laser light. The laser light was first coupled to an optical fiber, then collimated, introduced into the inset, and focalized on the sample. The luminescence was collimated and then coupled to an optical fiber (outside the cryostat) which guided the PL light to the spectrometer. The lens L3 (see Figure 1) was of crystalline quartz and served also as a cold filter cutting-off thermal radiation generated by room-temperature parts of the inset. PL spectra were continuously measured one after another during very slow scans of the magnetic field. The time of measurement of one spectrum was short enough (typically, 9 s) to allow for an increase of *B* by only 0.05 T which introduced only a negligible distortion in the data.

Transmission spectroscopy at the far-infrared was carried out with a Fourier spectrometer analyzing a signal from a bolometer placed just below the sample. In those measurements, the sample was cooled to 4.2 K with an exchange helium gas and placed in a center of a superconducting magnet. Thus, the conditions for far-infrared and PL spectroscopy were identical. Fourier spectra were taken at every 0.25 T. Raman scattering spectroscopy was carried out with samples kept at room temperature in the backscattering geometry, under no magnetic field. A HeNe laser (λ = 632.8 nm) was used which did not cause any strong PL and allowed to register spectra with a negligible background.

## 3 Results and Their Analysis

**Figure 2:**
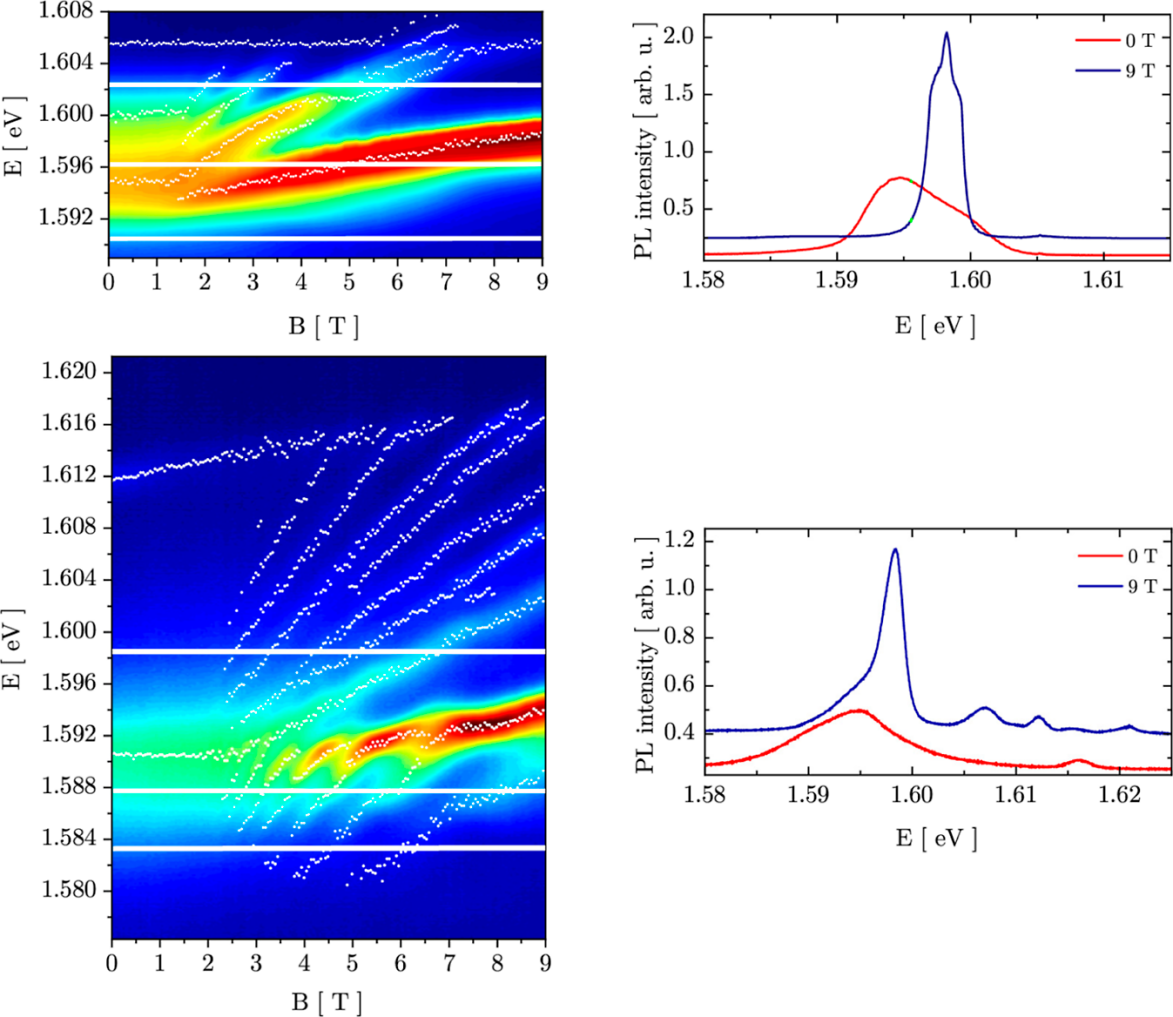
False-color PL maps for SQW (left, upper panel) and MQW (left, lower panel) samples measured at σ^–^ polarization. The PL intensity is presented in a logarithmic scale. White bars correspond to energy “slices” for which PL intensity was calculated as a function of *B* and are presented in Figure 3. White points show positions of maxima in spectra. Spectra measured at 0 and 9 T for both samples are shown in the right column and are vertically shifted for better presentation of PL from the second electrical sub-band at about 1.605 and 1.617 eV in the case of SQW and MQW samples, respectively, at 9 T.

False-color maps of the PL of both samples are shown in Figure 2. Comparing PL results for σ^–^ and σ^+^ polarizations, we observed a very small degree of polarization and practically no energy shift between the lines observed at different polarizations. For this reason, we are presenting and analyzing PL results for σ^–^ polarization only and neglect the spin splitting in the analysis. In PL measurements carried out on a sample similar to the SQW sample studied in the present paper, a spin splitting was observed.^20^ However, we notice that the widths of the lines of spectra presented in ref (20) are much narrower than those presented in Figure 2, and the experiment in ref (20) was carried out at a temperature equal to 85 mK which makes a drastic difference with respect to 4.2 K of the present study if the occupation of close-lying spin-split levels is considered.

The analysis of spectra is based on a technique presented by Babiński et al.,^18,19^ which relies on analyzing the intensity of luminescence in narrow-energy ranges (“slices”) as a function of magnetic field *B*. In this approach, it is assumed that the energy of electron and hole Landau levels (with a number *n*_e,h_ = 0, 1, 2...) changes according to *E*_c_ = *E*_0_ + (*n*_e_ + 1/2)*ℏeB*/*m*_e_ and *E*_h_ = −(*n*_h_ + 1/2)*ℏeB*/*m*_h_, with *m*_e_ and *m*_h_ being effective electron and hole masses, respectively. Then, the energy of transition at a given *B* is equal to *E*_0_ + (*n* + 1/2)*ℏeB*/m*, where 1/m* = 1/*m*_e_ + 1/*m*_h_. It is assumed here that the selection rule *n*_e_ = *n*_h_ ≡ *n* holds for an interband transition between the conduction and valence band Landau levels. If one considers a certain energy value *E*, then magnetic fields at which the transition energy coincides with *E* are given by solutions of the equation *E* = *E*_0_ + (*n* + 1/2)*ℏeB*_n_/m*, and then subsequent values of magnetic fields *B*_n_ are such that the separation of their inverse is given by
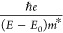
In other words, these values of magnetic fields are periodic in *B*^–1^, which means that the frequency at which they appear is given by *f*(*E*) = (*E* – *E*_0_)m*/(*ℏe*).

**Figure 3:**
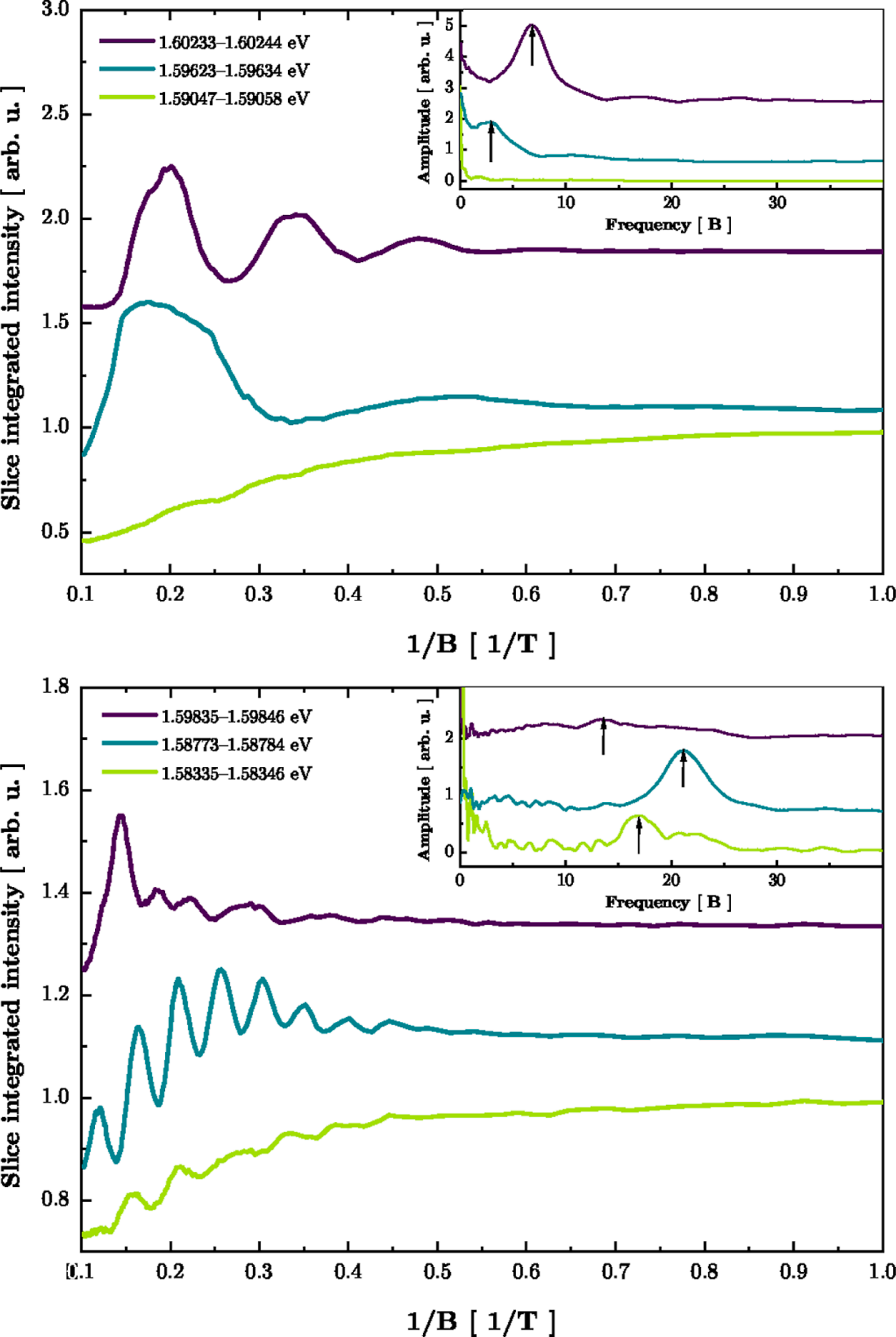
Intensity of “slices” of spectra for SQW (top) and MQW (bottom) samples measured at σ^–^ polarization. The legend shows the position of the slice (see white bars in Figure 2 for comparison). The width of each slice was equal 0.11 meV. Insets show a Fourier transform of traces presented in the main figure, calculated after subtraction of the background. Arrows show positions of peaks that are considered in further analysis in Figure 4.

In the above formulas, *E*_0_ denotes the separation between the ground states of electrons and holes taking part in the recombination process. In the case of an ideal (i.e., fluctuation-free) undoped QW, *E*_0_ is equal to the energy separating the ground states of electrons and holes in the QW, including an exciton correction. In QWs containing free electrons, *E*_0_ can be additionally influenced by screening, an electric field resulting from the presence of charges, and localizing potentials. In Figure 3, we present examples of PL intensity calculated for “slices” of PL spectra positioned at the energy indicated by white bars in Figure 2.

**Figure 4:**
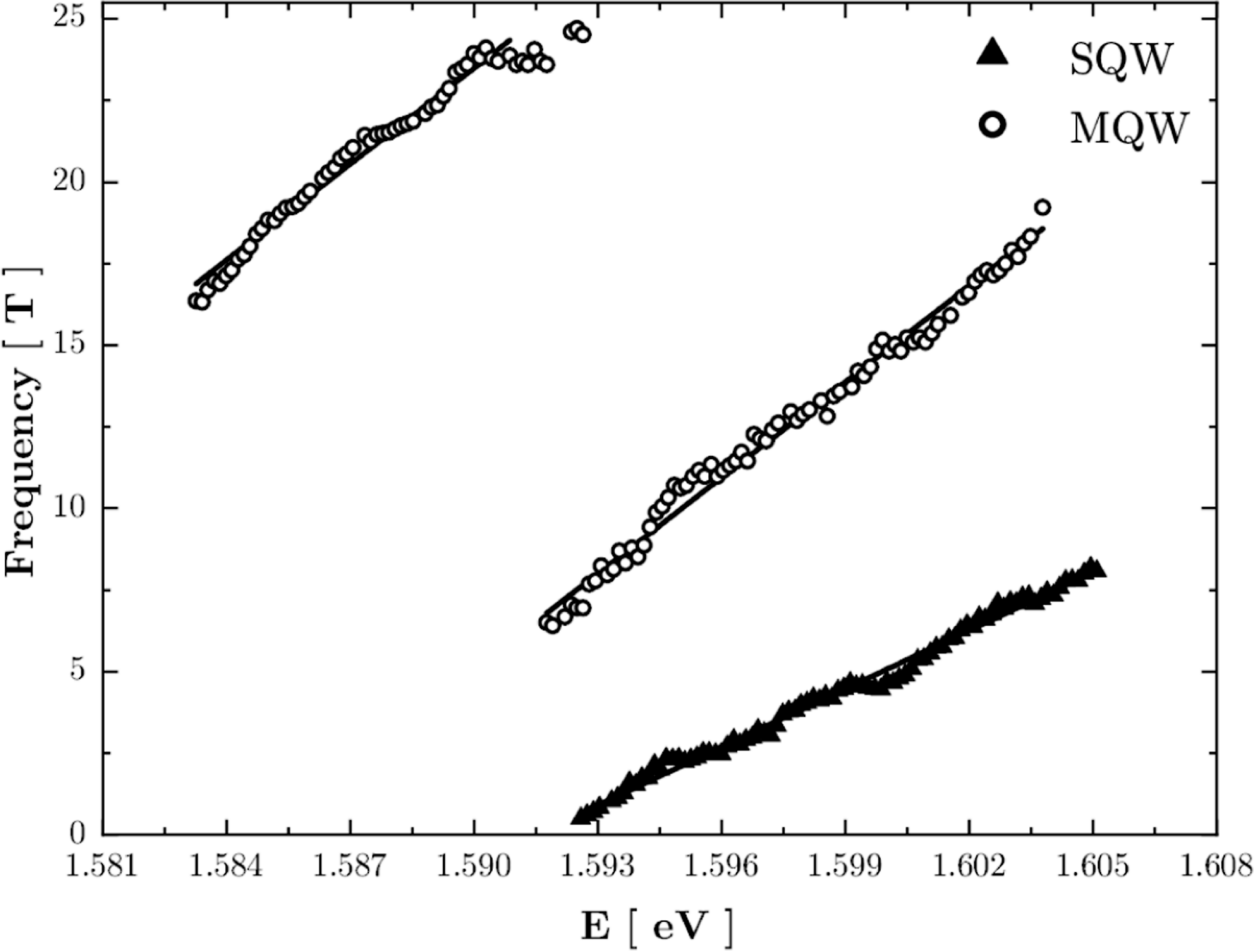
Frequency of oscillations of PL intensity as a function of the energy position of the slice. Triangles: SQW; circles: MQW. The plot contains points which could be unambiguously determined from the Fourier spectra. For this reason, they are restricted to a narrower energy range than the spectra shown in Figure 2.

As one can observe, the resulting plots are consistent with the presented reasoning, showing periodic oscillations in 1/*B*. Determination of the frequency *f*(*E*), for all values of *E* within the PL spectral range, was done by calculating a Fourier transform of oscillatory parts of the curves, showing a slice-integrated intensity as a function of 1/*B*. A few examples of the slice’s intensity vs 1/*B* are shown in Figure 3, together with their Fourier transform. Since the shape of the oscillatory part of the curves is complicated, Fourier transform shows a fundamental frequency with its harmonics, which were, however, not included in a graphical presentation of results in Figure 4. The position of the peak corresponding to the fundamental frequency could be determined unambiguously by inspection of the evolution of Fourier spectra, in spite of the fact that the choice of the main peak in Fourier spectra presented in Figure 3 could seem not evident in some cases.

Results of the Fourier analysis are collected in Figure 4. The figure also shows straight lines which were fitted to the data. The slope of the lines is clearly different for the two samples and leads to m* = (0.0689 ± 0.0007)*m*_0_ and m* = (0.113 ± 0.001)*m*_0_ in the case of SQW and MQW samples, respectively (*m*_0_ is the mass of electrons).

## 4 Discussion

The results plotted in Figure 2 show differences between the spectra of the SQW and MQW samples. In the first case, we observe a main peak split into Landau levels, and PL spectra cover approximately the range from 1.592 to 1.605 eV. In the case of MQW, the spectral range of PL is broader—from about 1.58 to 1.615 eV. A difference in the lower bound is caused by Landau-like maxima which are visible in the low-energy part of the MQW PL map in Figure 2. This part of the MQW PL spectra leads to points which are presented in the 1.583–1.592 eV range in Figure 4. As one can determine from Figure 4, the energy separation between two branches of MQW data (circular points) is equal to 18.8 ± 0.1 meV, i.e., 152 ± 8 cm^–1^. We did not observe any signature of a similar low-energy branch in the PL spectra of the SQW sample.

A substantial difference between the two samples is the width of the spacer (20 nm in SQW versus 10 nm in MQW) with the same doping level. A resulting higher concentration *n*_c_ in the MQW sample leads to a broader PL spectrum and clearly visible luminescence from the second electrical sub-band. This means that the Fermi level is close to the second electrical sub-band in the MQW sample. A rough estimate of *E*_F_ = 26 meV, based on the spectrum at *B* = 0 (the difference between the position of a small maximum at 1.611 and 1.585 eV), gives the electron concentration *n*_c_ = *m*_e_*E*_F_/(π*ℏ*^2^) = 1.2 × 10^12^ cm^–2^, which is comparable to 1.0 × 10^12^ cm^–2^ determined from transport measurements. On the other hand, signatures of the second electrical sub-band are barely visible in PL from the SQW sample. The value of 1.585 eV used above is the point at which extrapolation of the MQW line crosses the energy axis at a frequency equal to zero in Figure 4. This corresponds to an infinite period of oscillations in Figure 3, i.e., to zero carrier concentration at this energy. The value of 1.585 eV thus corresponds to the energy separation of the lowest electron and hole levels. The same reasoning applied to the SQW sample gives the energy 1.592 eV.

In undoped QWs, the energy separation between the ground hole and electron levels is equal to the energy band of the well’s material increased by the confinement energy of the electron and hole and, in some cases, further modified by exciton corrections and localization energy. The band gap energy of CdTe at 4.2 K is equal to 1.606 eV.^21^ The difference between 1.606 and 1.585 eV results mainly from the presence of the electric field in the QW. A simple estimation of the electric field appearing between two planes with 10^12^ elementary (positive and negative) charges per cm^–2^ gives 1.8 × 10^7^ V/m. This electric field would create a difference of about 360 mV within the well of a width of 20 nm. The presence of electrons in the well decreases this value due to screening, but this effect is essential in the energy scale of several meV considered here, and in our opinion the presence of an electric field in the well is the major factor reducing the energy of emitted photons with respect to the bulk CdTe. A higher electric field in the case of MQW than in the SQW sample explains why the lower bound of PL spectra in the MQW sample is at a smaller energy.

In principle, the above-presented model allowed one to determine the effective mass of the hole taking part in the luminescence if that of the electron is known or vice versa. Far-infrared Fourier spectroscopy in the transmission mode allowed us to observe a cyclotron resonance of electrons in studied QWs and determine the electron effective mass equal to (0.1112 ± 0.0003)*m*_0_ and (0.1094 ± 0.0003)*m*_0_ for SQW and MQW samples, respectively. For the SQW sample, with m* given in Section 3, one gets *m*_h_ = (0.181 ± 0.005)*m*_0_ which is approximately equal to the effective mass of light holes in bulk CdTe. In spite of this coincidence, we do not claim that these are light holes which take part in PL in the SQW sample. The thing is that description of the Γ_8_ valence band in zinc-blende materials in magnetic fields requires a special numerical treatment which was presented in ref (22) for the case of a GaAs/AlGaAs heterostructure. The same symmetry of band wave functions in CdTe and in GaAs allows us to apply qualitatively the results of calculations presented in ref (22) in the case of the present paper. First, in a QW, wave functions of light and heavy holes are mixed, and the degree of mixing depends on the wave vector. Second, the dependence of the energy of holes’ Landau levels on the magnetic field is not perfectly linear although deviations from linearity are not very strong (see Figure 5 in ref (22)).

In the case of the MQW sample, the obtained value of m* = (0.113 ± 0.001)*m*_0_ is within less than 4% equal to the electron mass in this sample, which is a strong argument that holes participating in the PL in this sample are localized and do not contribute to m* by a Landau quantization but with a much smaller spin splitting. A similar coincidence of m* and *m*_e_ in the case of PL involving localized holes was observed by Babiński et al. in the case of (Ga, In)As QWs.^18,19^

**Figure 5:**
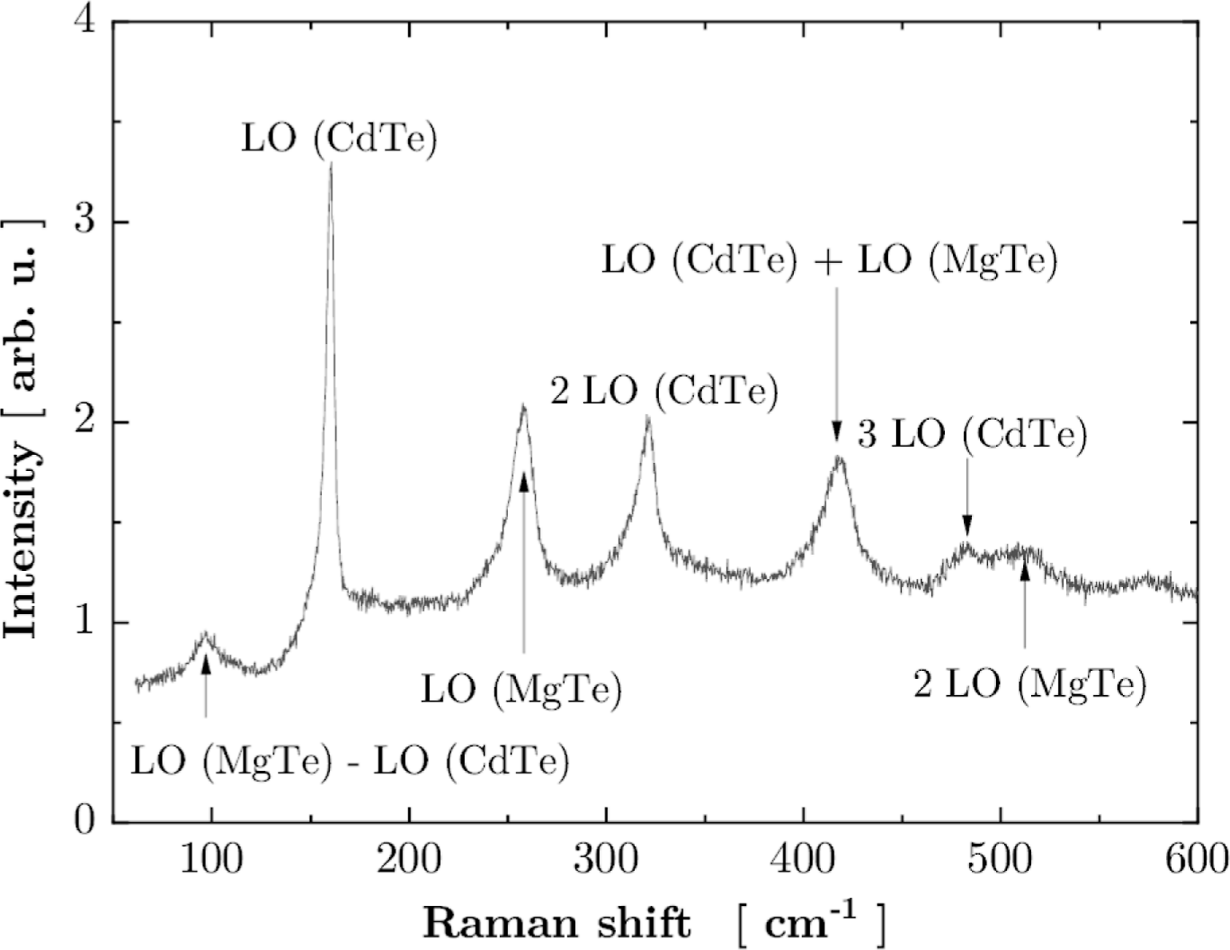
Raman spectrum measured on an MQW sample at 300 K with a 640 nm laser light. Identification of the lines is based on data presented in ref (23).

A first-guess interpretation of the low-energy part of MQW PL spectra is that they result from phonon replicas. A Raman spectrum presented in Figure 5 shows a CdTe LO phonon peak at 161 cm^–1^, which agrees—within the uncertainty—with the observed energy difference in Figure 4. However, this apparently smaller value (152 cm^–1^ vs 161 cm^–1^) can be attributed to the fact that in a disordered sample phonons can be localized, which means that they contain components with a nonzero wave vector. In such a situation, a decrease of LO phonon energy can be expected which, according to a shape of dispersion of the LO phonon, decreases with the increase of the wave vector. On the other hand, Raman spectroscopy in the backscattering configuration used in the present experiment probes phonons with the wave vector close to zero (point Γ of the Brillouin zone).

In conclusion, we carried out PL measurements on modulation-doped CdTe QWs with Cd_0.7_Mg_0.3_Te barriers. The samples were cooled to 4.2 K, and results were obtained in a magnetic field of up to 9 T. Two samples were studied: one with ten QWs and the other with one QW. An analysis of the intensity of PL as a function of *B* shows a qualitative agreement of data with a model based on Landau quantization of hole and electron levels in the case of one QW. In the case of multiple QW sample, the hole is localized, and the evolution of its quantum levels does not follow Landau quantization. Essential differences in the PL spectra were observed between the two samples. Interpretation of data should take into account mixing of heavy and light holes in the valence band of QWs, an influence of a built-in electric field, and also possible influence of fluctuations of the electrostatic potential resulting from the random distribution of impurities. In particular, these fluctuations could lead to a softening of the LO phonon mode necessary to explain phonon replicas in the PL of the multiple QW sample.
